# Sensorimotor gating, cannabis use and the risk of psychosis

**DOI:** 10.1016/j.schres.2015.02.017

**Published:** 2015-05

**Authors:** T. Winton-Brown, V. Kumari, F. Windler, A. Moscoso, J. Stone, S. Kapur, P. McGuire

**Affiliations:** a Department of Psychosis Studies, Institute of Psychiatry, Kings College London, United Kingdom; b Department of Psychology, Institute of Psychiatry, Kings College London, United Kingdom; c Center of Advanced European Studies and Research, Bonn, Germany; d Department of Child and Adolescent Psychiatry, Hospital de D. Estefânia Lisboa, Portugal

**Keywords:** Sensorimotor gating, Psychosis, Prepulse inhibition, Cannabis

## Abstract

Sensorimotor gating, measured as the modification of eye blink startle reflexes to loud acoustic stimuli by quieter preceding stimuli, is altered in those with psychosis, their relatives and those at high clinical risk for psychosis. Alterations have also been shown in cannabis users, albeit to a lesser extent, and cannabis is a known risk factor for the onset of psychosis in clinically and genetically susceptible individuals.

We examined the interaction between clinical risk for psychosis and cannabis use on sensorimotor gating, both Prepulse Inhibition (PPI) and Prepulse Facilitation (PPF). We tested PPI and PPF in participants with an At Risk Mental State (ARMS) for psychosis and a matched control group. Both groups included a proportion of subjects who had recently used cannabis, as confirmed by urinary drug screening (UDS) on the day of testing. We found that ARMS participants showed reduced PPF and PPI relative to controls, the latter driven by a group by cannabis use interaction, with recent use reducing PPI in ARMS participants but not in controls. When the analysis was limited to UDS-negative participants there was significantly reduced PPF in ARMS subjects relative to controls, but no differences in PPI. Within the ARMS group reduced sensorimotor gating, measured by both PPI and PPF, related to reduced overall level of function.

Cannabis use in clinical high risk individuals may increase the risk of psychosis in part through worsening PPI, while PPF is altered in ARMS individuals irrespective of cannabis use. This develops our understanding of cognitive mechanisms leading to the experience of aberrant perceptual phenomena and the subsequent development of psychotic symptoms.

## 1 Introduction

Sensorimotor gating is thought to play a role in how organisms allocate limited cognitive resources within a sensorially rich environment. Measuring the eyeblink startle reflex to a strong sensory stimulus, or ‘pulse’, can be used to study aspects of sensorimotor gating by examining the effect of a relatively weak preceding ‘prepulse’ (PP). This PP modifies the extent of the startle that follows according to the delay between stimuli, the inter-stimulus interval (ISI). When the ISI is short, between 30 and 480 ms, the startle reflex to the pulse that follows is attenuated, a phenomenon known as prepulse inhibition (PPI); with a longer ISI, between 500 and 2000 ms, the startle reflex to the following pulse is augmented, known as prepulse facilitation (PPF). PPI and PPF may reflect distinct processes: PPI at short ISI is thought to represent primarily an automatic pre-attentive gating mechanism (Braff et al., 1992), while attentional modulation of PPI occurs with ISI greater than 100 ms (Braff et al., 2001). PPF may represent later stages of sensory processing such as generalized alerting, orientation and passive attention (Graham, 1975).

In patients with psychotic disorders, deficits in sensorimotor gating may lead to cognitive fragmentation disorganization and psychotic symptoms, but the stage at which processing is altered is unknown (Kapur, 2003). Deficits in PPI in subjects with schizophrenia are well established (reviewed in Braff et al., 2001), and have been related to cognitive impairments and positive psychotic symptoms (Kumari et al., 2008c), and have been correlated with reductions in dorsolateral prefrontal, middle frontal and orbital/medial prefrontal volume (Kumari et al., 2008b). PPI deficits have also been reported in people with schizotypal (Cadenhead et al., 2000; Cadenhead, 2011) and psychosis-prone personality traits (Swerdlow et al., 1995a; Kumari et al., 2008a), and in the relatives of people with schizophrenia (Cadenhead et al., 2000; Kumari et al., 2005). These data suggest that PPI deficits may be a marker of vulnerability for psychosis.

There have been several previous studies of PPI in people at clinical high risk for psychosis. Quednow et al. (2008) found diminished PPI in this group, whereas Cadenhead (2011) found no differences between high risk subjects and controls, but *increased* PPI in high risk subjects who later developed psychosis relative to that in subjects who did not. More recently both Ziermans et al. (2011) and De Koning et al. (2014) found diminished PPI in clinical high risk groups, the latter screening out drug using participants using urinary testing. Biomarkers of clinical outcomes in this group are of particular interest, as they may facilitate the stratification of high risk samples according to the likelihood that an individual will subsequently develop psychosis or recover (Fusar-poli et al., 2012). Studies of PPI in this group also have the advantage of being free of the potentially confounding effects of antipsychotic medication on PPI (Kumari et al., 2007), as clinical high risk subjects are often medication naive.

Although there have been several studies of PPI in relation to psychosis, there have been relatively few studies of PPF (reviewed in Kumari et al., 2004, Schiz Res, Appendix 1/2). Wynn et al. (2004) found reduced PPF in subjects with schizophrenia and their first degree relatives compared to controls. There have not been any studies of PPF in subjects at clinical high risk.

A large proportion of patients with psychotic disorders and subjects at high risk of psychosis use psychoactive substances, particularly cannabis. Cannabis use can induce acute psychotic symptoms and is associated with an increased risk of developing a psychotic disorder (Arseneault et al., 2004; Moore et al., 2007). Little is known of the effects of substance use on PPI or PPF in either clinical or healthy samples, and the importance of UDS screening is well known (Swerdlow et al., 1995b). One study found PPI deficits in cannabis-using healthy controls only in actively attended to trials (Kedzior and Martin-Iverson, 2006)—in these attentional modulation paradigms participants are instructed to actively attend to prepulse and pulse sounds, compared to passive attention designs where no such direction is given. Similar findings emerged from a later study that compared cannabis using and non-using subjects with schizophrenia alongside healthy controls (Scholes-Balog and Martin-Iverson, 2011). Administering cannabinoids during adolescence to mice reproduced PPI deficits and several other markers of schizophrenia, (Gleason et al., 2012) and these were reversed by antipsychotic treatment (Nagai et al., 2006).

In the present study we set out to examine both PPI and PPF of the acoustic startle reflex in medication-free subjects with an At Risk Mental State for psychosis (Yung et al., 2005). They were compared with demographically- and geographically-matched healthy controls, and urinary drug screening was used to test for cannabis and other psychoactive substances. Our main hypothesis was that ARMS subjects would show PPI and PPF deficits relative to controls. A secondary hypothesis was that the findings would be modulated by cannabis use.

## 2 Methods

### 2.1 Recruitment

27 ARMS participants were recruited from Outreach And Support in South London (Fusar-Poli et al., 2013), a clinical service for the treatment of people at high risk of psychosis. At intake they were assessed by a psychiatrist using the Comprehensive Assessment of At Risk Mental States (Yung et al., 2005), and ARMS status was confirmed by consensus at multidisciplinary team meeting. All patients were antipsychotic naïve.

27 healthy control (HC) participants were recruited from the same geographical area, from the friends of the ARMS participants and via local advertisements. Control participants were excluded if they had a personal or family history of neurological or psychiatric disorder.

Written informed consent was obtained, and the local Research Ethics Committee approved the study protocol. Participants received compensation for their time and travel.

Prior to testing, all participants were assessed by a psychiatrist (TWB) and clinical scales were administered as follows: Hamilton Anxiety and Depression rating scales (Hamilton, 1960, 1959), Comprehensive Assessment of At Risk Mental States (CAARMS (Yung et al., 2005), and Peters Delusion Inventory (PDI Peters et al., 2004). Predicted IQ was estimated using the National Adult Reading Test (NART Nelson, 1991). Around half of the participants also participated in a separate session as part of another study where the history of substance use and their overall level of use for each substance was quantified on a scale of 0–4 (0 = never; 1 = experimental use, has tried occasionally, 2 = occasional use, has tried small quantitates from time to time; 3 = moderate use, has used small quantities regularly or large quantities occasionally; 4 = severe use, has frequently used large quantities, Table 2).

### 2.2 Protocol

A commercially available human startle response monitoring system (Mark II, SR-Lab, San Diego, California) was used to generate and deliver the acoustic stimuli, and to record and score the electromyographic (EMG) activity for 250 ms starting from the onset of the acoustic startle stimulus. Acoustic stimuli were presented to participants binaurally through well-sealed headphones (Telephonics TDH-39P). The pulse-alone stimulus was a 40-ms presentation of 114-dB (A) white noise and the prepulse stimulus a 20-ms presentation of 85-dB (A) white noise, both over 70-dB (A) continuous back-ground noise. The noise levels were calibrated using the continuous noise, and checked and re-calibrated on a monthly basis.

The session began with a 5 min acclimatization period consisting of 70 dB(A) continuous white noise. During the experiment, participants received four blocks of 21 trials each, after an initial pulse-alone trial; each block consisted of 3 pulse alone (PA) trials, 3 prepulse alone (PP) trials, 3 prepulse trials with a 30-ms prepulse-to-pulse (onset-to-onset) interval (PPI30), 3 prepulse trials with a 60-ms prepulse-to-pulse interval (PPI60), 3 prepulse trials with a 120-ms prepulse-to-pulse interval (PPI120), 3 prepulse trials with a 1000 ms prepulse-to-pulse interval (1000) and 3 prepulse trials with a 2000 ms prepulse-to-pulse interval (PPF2000). Trials were presented to participants in a pseudorandom order with a mean inter-trial interval of 15 s (range 9–23 s). The experiment lasted for 25 min, including the acclimation period.

The experimental procedures for recording and scoring the startle reflexes have been described in detail previously (e.g. Kumari et al., 2012). The eye blink component of the startle was indexed by recording EMG activity of the orbicularis oculi muscle directly beneath the right eye, using two miniature silver/silver chloride electrodes. Recorded EMG activity was band-pass filtered at 50-Hz, as recommended by the SR-Lab. The EMG data were first inspected on a trial-to-trial basis offline, then scored using the analytic program of this system for response amplitude (in arbitrary analogue-to-digital units; one unit = 2.62 μV) and latencies to response onset and peak. Responses were rejected if the onset and peak latencies differed by more than 95 ms, or when the baseline values shifted by more than 50 units (6.59% of trials). Noisy recordings, indicated by a high number of rejected trials (> 30%), were rejected outright; this led to 3 ARMS subjects and 4 HC subjects being excluded from analysis, leaving 23 HC and 24 HC subjects included in the final analysis.

PPI and PPF were computed for each participant separately for each trial type and block. PPI was calculated as (a − b / a) × 100, where “a” = pulse-alone amplitude and “b” = amplitude over prepulse trials. PPF was the inverse calculation: (b − a / a) × 100. Percent of PPI/PPF, rather than absolute amount (i.e. arithmetic difference between pulse-alone and prepulse trials), was used since this procedure reduces the influence of individual differences in startle responsiveness (Csomor et al., 2008). Psychophysiological data were scored blind to diagnosis and group membership.

Participants were told that the purpose of the experiment was to measure their reaction to a number of noise-bursts; no instruction was given on whether to attend or ignore them. They were asked to keep their eyes open during the experiment. Participants who smoked tobacco were not excluded, but they were not admitted to the testing suite until at least 30 minutes after their last cigarette, to minimize the potential for effects of recent nicotine intake or withdrawal on PPI or PPF (Postma et al., 2001; Swerdlow et al., 1996).

### 2.3 Analysis

Group differences in demographic and clinical measures were compared using chi-squared or paired *t*-tests.

Habituation over four blocks of pulse-alone trials was tested by entering the response amplitude of these trials into a repeated measures analysis of variance (ANOVA) with block as a within subject variable and group (HC, ARMS) as a between subject variable. Subsequent analyses used the mean measure across all blocks but also examined effects within the first block alone.

Separate analyses were undertaken for PPI and PPF. To examine group differences, PPI/PPF (%) scores were subjected to a 2 (Group: HC, ARMS) × 3/2 (Trial type: 30-ms, 60-ms and 120-ms prepulse trials for PPI, 1000-ms and 2000-ms for PPF) ANOVA, with group as a between-subjects and trial-type as a within-subject factor. This was followed by an ANCOVA to rule out confounding effects of gender (Aasen et al., 2005; e.g. Swerdlow et al., 1996) and smoking status.

To examine the effects of illicit substances, the analyses were repeated adding current cannabis use (UDS positive) as an additional between-subject factor. ANCOVAs were then conducted to rule out confounding effects of smoking and gender. Significant main effects were explored with planned post hoc pairwise comparisons using Tukey's Least Significant Difference measure. If the UDS was positive for substances in addition to cannabis (cocaine and amphetamine), these analyses were then repeated excluding these subjects.

In order to test relationships with clinical parameters, mean PPI at 60 ms and PPF at 1000 ms were testing using Spearman's correlations with total CAARMS positive score, Hamilton-A, Hamilton-D and Global Assessment of Functioning (GAF) score.

## 3 Results

There were no differences between groups on demographic measures (Table 1) or on self-reported substance use history (Table 2). As expected, all clinical measures were significantly greater in the ARMS group (Table 1). There were 6 ARMS and 5 HC subjects who tested positive for cannabis on the UDS. One of these ARMS subjects also tested positive for cocaine, and another also tested positive for cocaine and amphetamine. There were no differences on demographic or clinical measures between UDS positive and negative subjects within either group.

## 4 Habituation

**Fig. 1:**
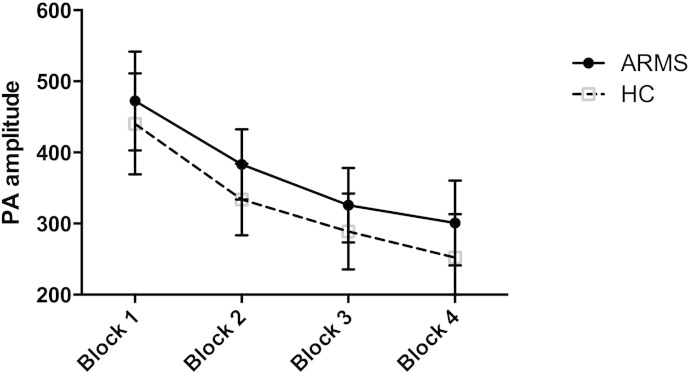
Significant habituation across blocks, error bars represent +/− 1 SEM.

There was a significant effect of block on startle amplitude to pulse alone trials, which decreased over subsequent blocks (Fig. 1, *F* = 12.9 *df* = 3, *p* < 0.0001), with no effect of group (*F* = 0.32, *df* = 1, *p* = 0.57) or group × block interaction (*F* = 0.027, *df* = 1, *p* = 0.871). To allow for this habituation in subsequent analyses we re-examined effects found across all blocks in the first block alone.

## 5 Startle reactivity

Startle reactivity was calculated by measuring the mean amplitude of the response to the pulse alone trials in the 4 blocks. An initial ANOVA was performed with block as within subject factor and group as a between subject factor. There was a significant effect of block (*F* = 12.882 *df* = 3 *p* < 0.001) but no group × block interaction. When substance use was added as an additional between subject factor this was not altered and there was no main effect of substance use or group × substance use interaction.

We then re-examined startle reactivity to the pulse alone trials from the first block by performing a univariate ANOVA with group and substance use as between subject variables. There was no effect of substance use or group, and no group × substance use interaction.

**Fig. 2:**
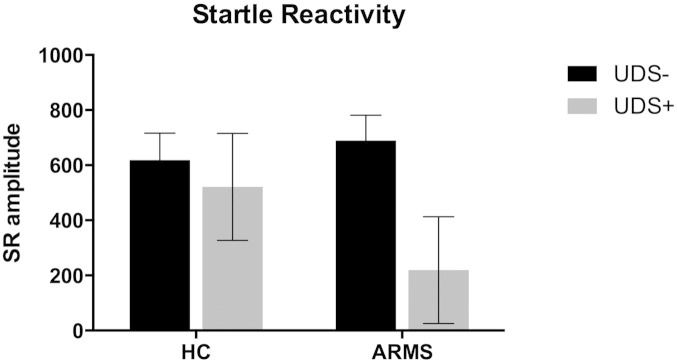
Amplitude of reaction to the first pulse by group and drug.

Finally we re-examined startle reactivity to the first pulse alone trial only. There was a trend to an effect of substance use (*F* = 3.468 *df* = 1 *p* = 0.07): those positive on UDS had reduced amplitude of startle reactivity to the first pulse alone trial in both groups but there was no main effect of group or group by drug interaction (Fig. 2, Supplementary Table 2). To account for this we added startle amplitude to the first pulse alone trial as an additional covariate in the PPI and PPF analyses where drug use was included as a between subject factor.

## 6 PPI

The ANOVA of mean PPI with trial-type as a within subject factor, and group as a between subject factor revealed no main effect of group or group × trial type interaction. Adding gender and smoking as covariates did not alter this result. However, when current substance use (UDS positive) was added as an additional between-subject variable, there was a significant group × substance use interaction (*F* = 4.478, *df* = 1 error *df* = 43 *p* = 0.04). This interaction remained significant when the substance effects were restricted to those of cannabis alone (by excluding the 2 subjects who were also positive for cocaine, *F* = 5.01 *df* = 1 error *df* = 41 *p* = 0.031), and when smoking (group × substance use interaction: *F* = 4.361 *df* = 1 error *df* = 42 *p* = 0.043, effect of smoking: *F* = 0.107 *df* = 1 error *df* = 42 *p* = 0.745) and gender (group × substance use interaction: *F* = 4.521 *df* = 1 error *df* = 42 *p* = 0.039, effect of gender: *F* = 7.97 *df* = 1 error *df* = 42 *p* = 0.007) were added as covariates. Adding overall mean startle response amplitude reduced the significance of the interaction (group × substance use interaction: *F* = 3.274 *df* = 1 error *df* = 42 *p* = 0.018, effect of startle response *F* = 0.511 *df* = 1 error *df* = 37 *p* = 0.497).

**Fig. 3:**
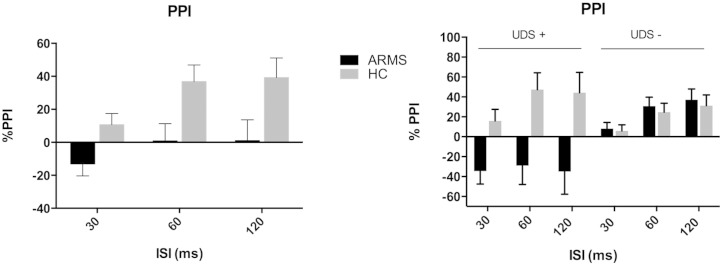
A) Mean 1st block PPI type by group, and 3B) by group and cannabis use. Error bars represent +/− 1 SEM.

When these analyses were repeated using PPI from the first block alone, the ANOVA of 1st block PPI with trial type as a within-subject factor, and group and drug as between-subject factors revealed a significant main effect of group (*F* = 5.84 *df* = 1 error *df* = 43 *p* = 0.02), and a significant group × drug interaction (*F* = 8.37 *df* = 1 error *df* = 43 *p* = 0.006): ARMS subjects had reduced PPI overall compared to controls, and in controls cannabis use was associated with increased PPI, whereas the opposite applied in ARMS subjects. Both these findings were stronger when the substance use effects were restricted to cannabis alone (group effect *F* = 8.994 *df* = 1 error *df* = 41 *p* = 0.005, Fig. 3A, Supplementary Table 3A, group × substance use interaction: *F* = 11.88 *df* = 1 error *df* = 41 *p* = 0.001, Fig. 3B, Supplementary Table 3B). They also remained significant when gender (group effect *F* = 4.805 *df* = 1 error *df* = 42 *p* = 0.034, group × substance use interaction *F* = 8.149 *df* = 1 *p* = 0.007, effect of gender *F* = 2.595 *df* = 1 error *df* = 42 *p* = 0.115) smoking (group effect *F* = 4.636 *df* = 1 error *df* = 42 *p* = 0.037, group × substance use interaction *F* = 8.389 *df* = 1 *df* = 42 *p* = 0.006, effect of smoking *F* = 1.812 *df* = 1 error *df* = 42 *p* = 0.185) and mean 1st block startle response amplitude were added as covariates to these analyses (group effect *F* = 5.832 *df* = 1 *df* = 42 *p* = 0.02, group × substance use interaction *F* = 4.774 *df* = 1 error *df* = 42 *p* = 0.035, effect of startle response *F* = 11.176 *df* = 1 error *df* = 42 *p* = 0.002).

## 7 PPF

Identical analyses were conducted for PPF. The ANOVA of mean PPF with trial-type as a within subject factor and group as a between subject factor revealed a trend towards a main effect of group (*F* = 3.317 *df* = 1 error *df* = 40 *p* = 0.076), with a reduction in PPF in ARMS subjects. This was not altered by adding mean startle amplitude as a covariate (group effect *F* = 2.896 *df* = 1 error *df* = 39 *p* = 0.097, effect of startle response *F* = 1.429 *df* = 1 error *df* = 39 *p* = 0.239) and was strengthened by adding gender as a covariate (group effect *F* = 5.104 *df* = 1 error *df* = 39 *p* = 0.03, effect of gender *F* = 4.353 *df* = 1 error *df* = 39 *p* = 0.044) and by adding smoking additionally (group effect *F* = 4.539 *df* = 1 error *df* = 39 *p* = 0.04, effect of smoking *F* = 0.287 *df* = 1 error *df* = 38 *p* = 0.595). Adding current substance use as an additional between subject-factor did not reveal a significant group × substance use interaction.

**Fig. 4:**
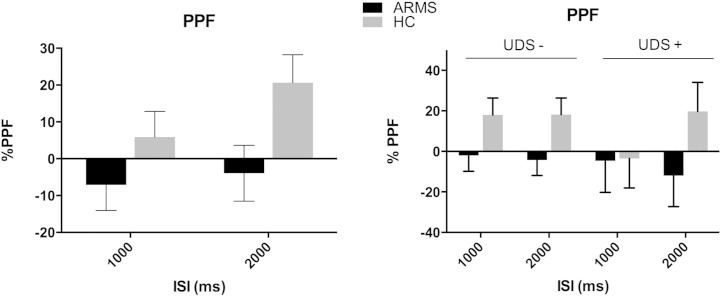
A) Mean 1st block PPF type by group, and 4B) by group and cannabis use. Error bars represent +/− 1 SEM.

We repeated the above analyses using PPF from the first block alone. Again there was a trend towards an effect of group (*F* = 3.65 *df* = 1 error *df* = 40 *p* = 0.063), PPF was reduced in ARMS relative to control participants. This effect was not altered by adding mean startle amplitude from the first block (group effect *F* = 3.079 *df* = 1 error *df* = 39 *p* = 0.087, effect of startle response *F* = 0.524, *df* = 1 error *df* = 39 *p* = 0.524) but was strengthened by adding gender as a covariate (group effect *F* = 4.454 *df* = 1 error *df* = 39 *p* = 0.041, effect of gender *F* = 1.391 *df* = 1 error *df* = 39 *p* = 0.245) and by adding smoking additionally (group effect *F* = 4.507 *df* = 1 error *df* = 38 *p* = 0.04, effect of smoking *F* = 0.155 *df* = 1 error *df* = 38 *p* = 0.696, Fig. 4A, Supplementary Table 4A). Adding current substance use as an additional between subject-factor did not reveal a significant group × substance use interaction (Fig. 4B, Supplementary Table 4B).

## 8 PPI and PPF in UDS negative participants

We repeated the main analyses after excluding all subjects with a positive urinary drug screen. In UDS negative subjects there was no main effect of group for mean or 1st block PPI, but there was a trend for an effect of group for mean PPF (*F* = 3.023 *df* = 1 error *df* = 30 *p* = 0.092) which was stronger in the 1st block (*F* = 3.94 *df* = 1 error *df* = 30 *p* = 0.056), and significant when sex and smoking were included as covariates (group effect *F* = 5.360 *df* = 1 error *df* = 30 *p* = 0.028, effect of gender *F* = 1.323 *df* = 1 error *df* = 28 *p* = 0.260, effect of smoking *F* = 0.772 *df* = 1 error = 28 *p* = 0.387).

## 9 Relationship of PPF and PPI to clinical measures

Within the ARMS, there were no significant correlations between mean PPI at 60 ms and mean PPF at 1000 ms and CAARMS positive, PDI, SPQ-B, Ham-A or Ham-D scores. Significant correlations were found between overall function, as measured by the GAF, and PPI at 60 ms (*ρ* = − 0.502, *p* = 0.021 (and PPF at 1000 ms (*ρ* = 0.529, *p* = 0.024).

## 10 Relationship of PPF and PPI to cannabis use

Within those who tested positive for cannabis use, there was no correlation between the between mean PPI at 60 ms and mean PPF at 1000 ms self reported frequency of cannabis use.

## 11 Discussion

We tested PPI and PPF of the eyeblink startle reflex to acoustic stimuli in participants at high clinical risk for psychosis and a matched control group. Both groups included a proportion of subjects who had recently used psychoactive substances, primarily cannabis, as confirmed by urinary drug screening on the day of testing. Because there was significant habituation across blocks we tested 1st block trials alone as well as all trials together. In the 1st block ARMS participants showed reduced PPI relative to controls, and this appeared to be driven by a group by cannabis use interaction, with recent use reducing PPI in ARMS participants but not in controls. When the analysis was limited to UDS-negative participants there was significantly reduced PPF in ARMS subjects relative to controls, but no differences in PPI. PPI and PPF both related to overall function in the ARMS as measured by the GAF.

A reduction in PPI in ARMS participants is consistent with the type of deficit evident in participants with psychotic disorders (Parwani et al., 2000; Wynn et al., 2004), their relatives (Kumari et al., 2005), and in people with psychosis-prone personalities (Kumari et al., 2008a). This suggests that PPI may represent a biomarker for vulnerability to psychosis, and adds weight to evidence of the validity of the Attenuated Psychosis Syndrome that has been much debated in DSM-V (Ruhrmann et al., 2010). However, our findings also suggest that cannabis use has differential effects on PPI in ARMS and controls; when cannabis users were excluded from the analyses PPI in the ARMS group was normal. In previous studies of PPI where urine testing for recent substance use was not conducted, including in samples of psychotic subjects (Parwani et al., 2000) and in clinical (Quednow et al., 2008), and genetic (Wynn et al., 2004) high risk samples, this raises the possibility that these findings may have been confounded by the effects of cannabis use. A further possibility is that those who were positive for cannabis on urinary testing are ‘true’ at risk for psychosis participants whose altered PPI is part of the prodrome of later psychosis. The present cross sectional data are unable to answer this interesting possibility.

A differential effect of cannabis on PPI in high risk subjects is in line with evidence of the symptomatic effects of cannabis in this group (Henquet et al., 2005; van Os et al., 2010). It is also consistent with evidence that effects of cannabis use on psychotic symptoms and on the risk of developing a psychotic disorder are moderated by genetic risk for psychosis and specific polymorphisms (Caspi et al., 2005; Henquet et al., 2008).

A differential effect of cannabis attenuating PPI in high risk subjects is also consistent with evidence that chronic cannabis users have reduced PPI, although only in attended-to trials (Kedzior and Martin-Iverson, 2006), and that drug free chronic cannabis users have PPI comparable to controls (Quednow et al., 2004). The finding of increased PPI in cannabis positive controls meanwhile is similar to another recent study (Preller et al., 2013) and consistent with evidence that CB1 agonists similarly induced PPI increases in rodents (Long et al., 2010; Stanley-Cary and Harris, 2002).

It is plausible that some of the psychoactive effects of cannabis in susceptible individuals are via effects on sensorimotor gating, both at early and later stages: in rats, agonists to the CB1 receptor, the principal target of THC in the brain, reduce prepulse inhibition, reversed by haloperidol (Schneider and Koch, 2002), and modulate emotional associative learning and memory formation (Laviolette and Grace, 2006). In humans Delta-9-tetrahydrocannabinol (THC), the key psychoactive constituent of cannabis, alters neural responses during basic sensory processing (Winton-Brown et al., 2011) and attentional oddball processing (Bhattacharyya et al., 2012), the latter particularly in the striatum and prefrontal cortex (Bhattacharyya et al., 2012). Alterations in so called ‘salience processing’ which may incorporate both early and late sensorimotor gating (Winton-Brown et al., 2014), induced by cannabis in susceptible individuals, may lead to the detection and prominence of stimuli that should have been filtered out, and induction of psychotic symptoms (Bhattacharyya et al., 2012; Kapur, 2003).

Cannabis did not appear to differentially alter sensorimotor gating at a later stage, as indexed by PPF. PPF was however significantly reduced in ARMS participants who were UDS-negative, suggesting that there may be later stage gating abnormalities in people at high risk of psychosis. These are similar to the findings from a study that found that PPI in the siblings of patients with schizophrenia was not different from controls, but that PPF was significantly reduced (Wynn et al., 2004).

We did not find relationships between either PPI or PPF and positive psychotic symptoms, consistent with previous literature suggesting that gating deficits relate more to cognitive deficits and disorganization (Braff et al., 1999; Karper et al., 1996). We did however find relationships of PPI and PPF sensorimotor gating with overall function, similar to Swerdlow et al. (2006) in participants with schizophrenia.

Our study had a number of important limitations, including a modest sample size; the subsample of UDS positive participants was particularly small with 5 and 6 in control and UHR groups respectively. The findings should be thus regarded as preliminary and require replication in larger samples. A limited sample size may also have limited the study's power to detect PPI deficits in the ARMS group regardless of cannabis use. However given the marked effects of cannabis use on PPI, in this sample future studies should include urinary drug it may be useful to collect more detailed information on the pattern and experience of substance use.

## 12 Conclusions

In this sample of subjects at high clinical risk for psychosis, we found deficits in both early and late stage sensorimotor gating, with the former moderated by cannabis use. Further work is needed to uncover the neural basis of sensory gating deficits and how these relate to aberrant salience and the development of psychotic symptoms.

## Role of funding source

The Wellcome Trust supported TWB during this study and reimbursed subjects' travel and time.

## Contributors

Drs Winton-Brown, Stone, Kumari, Kapur and McGuire conceptualized the paper. Drs Winton-Brown, Moscoso and Windler collected the data. Dr Kumari supervised the data analysis. Dr Winton-Brown performed the data analysis and drafted the paper. All authors assisted with subsequent drafts and approved of the final draft.

## Conflict of interest

There are no relevant conflicts of interest.

## Figures and Tables

**Table 1:** Demographics and clinical measures of subjects included in final analysis.

	ARMS	HC	Statistic (*t* / *x*^2^) / *p*
*n*	24	23	-
Age—mean (SD)	22.0 (3.5)	23.5 (4.0)	1.39/ 0.172
Gender (F/M)	13/11	8/15	0.621/ 0.73
Smoker	13	8	0.181
UDS positive	6	5	0.792
Predicted IQ (NART)—mean (SD)	110 (9.5)	114 (11.4)	1.241/0.22
CAARMS—pos mean (SD)	7.62 (3.1)	0.8 (1.2)	9.5/ < 0.0001
Ham-A—mean (SD)	15.0 (6.9)	2.0 (3.2)	8.0/ < 0.0001
Ham-D—mean (SD)	14.5 (8.2)	1.6 (2.3)	7.1/ < 0.0001
SPQ-B—mean (SD)	13.3 (3.8)	4.6 (4.5)	7.43/ < 0.0001
PDI—mean (SD)	66.1 (39.5)	22.7 (23.5)	4.22/ < 0.0001
GAF—mean (SD)	54.9 (6.6)	81.9 (10.7)	7.76/ < 0.0001

UDS Urinary Drug Screen; NART National Adult Reading Test; CAARMS Comprehensive Assessment of At Risk Mental States; Ham-A Hamilton Anxiety Rating Scale; Ham-D Hamilton Depression Rating Scale; SPQ-B Schizotypy Personality Questionnaire Brief; PDI Peters Delusional Index; GAF Global Assessment of Functioning.

**Table 2:** Self reported substance use history.

	ARMS	HC	Statistic (*t* / *x*^2^) / *p*
Any illicit drugs—ever used? (y/n)	10/4	8/6	0.62/0.430
Cannabis—ever used? (y/n)	10/4	10/4	0.27/0.605
Cannabis—frequency of use (0–4)—mean (SD)	1.9 (1.6)	1.1 (1.2)	1.57/0.131
Amphetamine—ever used? (y/n)	5/9	2/12	1.71/0.190
Amphetamine—frequency (0–4)—mean (SD)	0.9 (1.3)	0.2 (0.4)	1.70/0.101
Cocaine—ever used? (y/n)	4/10	2/12	0.85/0.357
Cocaine—frequency (0–4)—mean (SD)	0.8 (1.5)	0.1 (0.4)	1.58/0.126
Ecstasy—ever used? (y/n)	4/10	3/11	0.19/0.663
Ecstasy—frequency (0–4)—mean (SD)	0.7 (1.2)	0.2 (0.4)	1.47/0.155
