# Supplementary material for: Sensorimotor gating, cannabis use and the risk of psychosis
Source: Schizophr Res. 2015 May;164(1-3):21–7. doi: 10.1016/j.schres.2015.02.017 (PMC4435667; doi:10.1016/j.schres.2015.02.017)
Supplement: Supplementary file 1 — Supplementary tables. [file mmc1.pdf]

Supplementary Table 1 - Habituation

	ARMS			HC		
Block	Mean	SEM	SD	Mean	SEM	SD
1	472.479	69.501	375.275	440.043	70.996	299.83
2	383.243	49.261	254.942	333.855	50.321	226.225
3	325.778	52.31	308.091	288.79	53.435	187.338
4	300.813	59.677	362.014	252.051	60.961	194.472

Supplementary table 2 - Initial Startle response

group	UDS	Mean	Std. Error	95% Confidence Interval	
				Lower Bound	Upper Bound
ARMS	positive	219.25	194.08	-173.644	612.144
	negative	689.944	91.49	504.733	875.156
HC	positive	521.25	194.08	128.356	914.144
	negative	618.875	97.04	422.428	815.322

Supplementary table 3A %PPI in first block

group	PPI (ms)	Mean	Std. Error	95% Confidence Interval	
				Lower Bound	Upper Bound
ARMS	30	-13.166	7.233	-27.772	1.441
	60	0.842	10.49	-20.344	22.027
	120	1.079	12.57	-24.307	26.465
HC	30	10.851	6.614	-2.507	24.209
	60	35.973	9.594	16.599	55.348
	120	37.777	11.496	14.561	60.993

Supplementary table 3B %PPI in first block

group	UDS	PPI (ms)	Mean	Std. Error	95% Confidence Interval	
					Lower Bound	Upper Bound
ARMS	positive	30	-34.504	13.084	-60.928	-8.08
		60	-29.044	18.977	-67.37	9.281
		120	-34.995	22.74	-80.92	10.93
	negative	30	8.172	6.168	-4.284	20.629
		60	30.728	8.946	12.661	48.794
		120	37.153	10.72	15.504	58.803
HC	positive	30	15.852	11.703	-7.783	39.486
		60	47.313	16.974	13.033	81.592
		120	44.296	20.34	3.219	85.372
	negative	30	5.851	6.168	-6.605	18.308
		60	24.634	8.946	6.567	42.701
		120	31.258	10.72	9.609	52.907

Supplementary table 4A %PPF in first block

group	PPF (ms)	Mean	Std. Error	95% Confidence Interval	
				Lower Bound	Upper Bound
ARMS	1000	-7.1	6.966	-21.202	7.002
	2000	-3.925	7.591	-19.292	11.442
HC	1000	5.903	6.966	-8.199	20.005
	2000	20.642	7.591	5.276	36.009

Supplementary table 4B %PPF in first block

group	UDS	PPF (ms)	Mean	Std. Error	95% Confidence Interval	
					Lower Bound	Upper Bound
ARMS	positive	1000	-4.62	15.583	-36.223	26.983
		2000	-11.961	15.291	-42.971	19.05
	negative	1000	-2.024	7.825	-17.895	13.847
		2000	-4.267	7.679	-19.84	11.307
HC	positive	1000	-3.491	14.599	-33.1	26.118
		2000	19.72	14.326	-9.334	48.774
	negative	1000	18.008	8.363	1.048	34.968
		2000	18.147	8.206	1.505	34.79
